# An efficient multi-factor authentication scheme based CNNs for securing ATMs over cognitive-IoT

**DOI:** 10.7717/peerj-cs.381

**Published:** 2021-03-02

**Authors:** Ahmed Shalaby, Ramadan Gad, Ezz El-Din Hemdan, Nawal El-Fishawy

**Affiliations:** Department of Computer Science and Engineering, Faculty of Electronic Engineering, Menouf, Menoufia, Egypt

**Keywords:** Iris recognition, Deep learning, Convolutional neural networks, Chaotic encryption, ATM, Cognitive IoT, Mobile application

## Abstract

Nowadays, the identity verification of banks’ clients at Automatic Teller Machines (ATMs) is a very critical task. Clients’ money, data, and crucial information need to be highly protected. The classical ATM verification method using a combination of credit card and password has a lot of drawbacks like Burglary, robbery, expiration, and even sudden loss. Recently, iris-based security plays a vital role in the success of the Cognitive Internet of Things (C-IoT)-based security framework. The iris biometric eliminates many security issues, especially in smart IoT-based applications, principally ATMs. However, integrating an efficient iris recognition system in critical IoT environments like ATMs may involve many complex scenarios. To address these issues, this article proposes a novel efficient full authentication system for ATMs based on a bank’s mobile application and a visible light environments-based iris recognition. It uses the deep Convolutional Neural Network (CNN) as a feature extractor, and a fully connected neural network (FCNN)—with Softmax layer—as a classifier. Chaotic encryption is also used to increase the security of iris template transmission over the internet. The study and evaluation of the effects of several kinds of noisy iris images, due to noise interference related to sensing IoT devices, bad acquisition of iris images by ATMs, and any other system attacks. Experimental results show highly competitive and satisfying results regards to accuracy of recognition rate and training time. The model has a low degradation of recognition accuracy rates in the case of using noisy iris images. Moreover, the proposed methodology has a relatively low training time, which is a useful parameter in a lot of critical IoT based applications, especially ATMs in banking systems.

## Introduction

In the domain of banking and commercial establishments, identity verification of ATMs’ users is very momentous. Verification needs high-security levels for personal information and privacy protection against prohibitive use. Currently, using a combination of a credit card and password is the most widespread method, but this technique has vulnerabilities such as credit card damage and fraud. One of the alternative solutions is applying biometric techniques (Bolle et al., 2004) on ATMs. This provides more efficient and reliable identification methods based on iris recognition. Iris is considered one of the most precise biometrics available today; because of its desirable characteristics (Bowyer & Burge, 2013). Implementation of an efficient iris recognition system—with a low probability of break-ins in critical IoT environments (like ATMs)—has many complexities (such as: securing the communication channels between ATMs and bank’s classification servers, and dealing with noisy iris captured). This may be because of noise interference with ATMs cameras or bad iris acquisition by users.

It is necessary—in an iris recognition system for ATMs—to classify all bank clients (Bishop, 2006). It is important to extract the best iris features that characterize the customer’s data; to facilitate the role of the classifier and reduce its complexity. Designing handcrafted feature extractors for the iris biometric becomes a complex and challenging task. Full knowledge of the nature and characteristics of the iris is needed, and it is of course not guaranteed to achieve a high accuracy rate. Deep learning (Jordan & Bishop, 2004; Haykin, 2008; Vinayakumar et al., 2019a), especially Convolutional Neural Networks (CNNs) (Courville, Goodfellow & Bengio, 2016), can give us a very good understanding of image data, without depending completely on any domain knowledge and handcrafted features.

Many researchers (Minaee, Abdolrashidiy & Wang, 2016; Alaslani & Elrefaei, 2018), who addressed the use of CNNs with iris traits used a pre-trained model of CNNs such as VGG-16 (VGG16 architecture, 2018), ResNet50 (ResNet architecture, 2019), and Inceptionv3 (Keras, 2020, keras.io). These pre-trained models are trained on a very large number of data classes that exclude iris classes themselves and use these models as a black box. So, using such pre-trained models as they are is not warranted for achieving high accuracy recognition rates because of the biometric information loss which was not used in the training stage of these models. Building a new CNN model, as proposed, needs a very careful selection of the number of kernels, the kernels’ dimensions, input image dimensions, learning rate, and other factors that affect the model recognition rate (Sriram et al., 2020). It also requires a large number of training and testing experiments in order to achieve the best architecture that has a higher recognition accuracy rate with relatively low training time (Vinayakumar et al., 2019b).

Many existing systems for iris recognition achieve a well-accepted recognition rate (Al-Waisy et al., 2018; Gaxiola, Melin & Lopez, 2010). But the majority of them deal with irises acquired by infrared or near-infrared cameras (Center for Biometrics and Security Research, 2020), which are completely unsuitable in the domain of ATMs. They mainly depend on usual light vision cameras.

Even with using iris biometric-based IoT environments such as ATMs, the communication channels are still a weak point in the overall system. Any penetration of these channels endangers the system. So iris encryption is crucial here, but conventional cryptography techniques like AES, RSA, and DES (Stallings, 2016; Menezes, Van Oorschot & Vanstone, 1996) are unsuitable for biometric data due to inseparable characteristics of biometric data like high correlation among adjacent pixels, high redundancy, etc. (Mehta, Dutta & Kim, 2016). The chaotic theory is favorable for encryption of biometric data, as it is very sensitive to initial conditions, pseudorandom in nature, and has high resistivity against system attacks (Dachselt & Schwarz, 2001).

In this article, the main contributions are:

- Propose an efficient full authentication system for ATMs based on a bank’s mobile application. The iris recognition depends on handcrafted deep CNN as a feature extractor, and a fully connected neural network (FCNN)—with Softmax layer—as a classifier.
- Provide a secure method to address the problem of hacking the iris template transmission over the communication channel between ATMs and the bank servers by protecting the iris using chaotic encryption.
- The proposed system is evaluated via various experimental test cases that will be captured using usual light vision cameras which make them suitable for ATMs like the two public datasets Phoenix (Dobeš et al., 2004, 2006; Dobeš & Machala, 2020) and UBIRIS. V1 (UBIRIS, 2020). The system is also evaluated via datasets acquired by near-infrared cameras such as the CASIA V4 dataset (Center for Biometrics and Security Research, 2020). Likewise, we study and discuss the effect of several kinds of noise on iris images. It is due to noise interference or bad acquisition or any other system attacks.

The rest of this article is organized as follows: related works are presented in “Related Work”. “Proposed System” presents the proposed iris recognition system. Next, the experimental results are presented in “Experimental Results”, and the discussion in “Discussion”. Finally, the conclusion of this article is presented in “Conclusion”.

## Related work

Several works have been addressed in the field of iris recognition. The researchers differ mainly in the combination of the methods used for feature extraction and classification. Many used handcrafted feature extractors to build their classification systems. Some works addressed the use of CNN as a feature extractor. Using handcrafted feature extractors to extract iris features—as we mentioned—needs full knowledge of the nature and characteristics of iris, and it is of course not guaranteed to achieve a high accuracy rate. Most of the researchers (Minaee, Abdolrashidiy & Wang, 2016; Alaslani & Elrefaei, 2018; Lozej et al., 2019) who addressed the use of CNNs to extract features in their works used pre-trained models like VGG-Net, Alex-Net, Inceptionv3, and LeNet-5. In that case, they lose a lot of information associated with the iris itself which decreases the accuracy of the recognition rate.

The authors in Lozej et al. (2019), proposed an iris recognition system, with the pre-trained model of Xception as a feature extractor and the Pre-trained model DeepLabV3+ with MobileNet as a classifier. They tested their model against CASIA Thousand dataset. They achieved a 97.46% accuracy of recognition rate, which is considered a relatively good recognition rate, but it could be better without using these generic pre-trained models. The authors in Minaee, Abdolrashidiy & Wang (2016), proposed an iris recognition system, with the pre-trained model of Visual Geometry Group at the University of Oxford (VGG-Net) as a feature extractor and a multi-class Support Vector Machine (SVM) algorithm as a classifier. They tested their model against the CASIA-Iris-Thousand dataset and get a 90% accuracy of recognition rate, which is considered a relatively moderate recognition rate due to using a pre-trained model and the loss of biometric information during training.

An iris recognition system is proposed in Singh et al. (2020), where the researchers used Integer Wavelet Transform (IWT) as an iris feature extractor and normalized Hamming distance as a classifier. The UBIRIS.v2 dataset was used for testing their model. They achieved a 98.9% accuracy of the recognition rate, which is considered a good recognition rate but the UBIRIS.v2 dataset is based only on one eye, which makes it less suitable for critical IoT applications. Another iris recognition system is proposed in Alaslani & Elrefaei (2018), where the authors used the pre-trained Alex-Net model as a feature extractor and a multi-class SVM algorithm as a classifier. They tested their model CASIA-Iris-Interval dataset and get 89% as the accuracy of the recognition rate, which is considered also a relatively moderate recognition rate because of using a pre-trained model.

The authors in Rana et al. (2019), used Discrete Wavelet Transform (DWT) with Principle Component Analysis (PCA), as a handcrafted method for extracting features and Support Vector Machine (SVM) as a classifier. They tested their model against CASIA-Iris-V4. They get 95.40% as the accuracy of the recognition rate. A proposed iris recognition system in Sundaram & Dhara (2011), with Haralick texture for extracting features and probabilistic neural networks (PNN) as a classifier. They tested their model against UBIRIS. V1. dataset, and get 97% accuracy of recognition rate, which is considered a Fairly good recognition rate. The authors in Dhage et al. (2015), used Discrete Wavelet Transform (DWT) and Discrete Cosine Transform (DCT), as a handcrafted method for extracting features and Euclidean Distance as a classifier. They tested their model against the Phoenix dataset. They have an average of 88.5% recognition rate. Also, their pre-processing stage does not include iris preprocessing operations of segmentation and normalization which affects their recognition rate.

Some authors (Gad et al., 2019), who addressed the use of iris biometric in IoT applications, do not apply any encryption algorithm for iris templates before transmission, and this drawback endangers the overall system. Most the of works (Mehta, Dutta & Kim, 2016; Bhatnagar & Wu, 2014; Liu, 2012), which addressed biometric chaotic encryption, focused only on security issues and did not give sufficient consideration to the problem of iris recognition. So, there is a lack of works, which addressed the problem of building efficient full authentication systems for IoT based applications that provide a secure method to address the problem of hacking the iris templates transmission over the communication channels.

Also, most of the researchers (Dhage et al., 2015; Bharath et al., 2014; Saminathan, Chakravarthy & Chithra Devi, 2015) who worked in iris recognition, their classification models are based only on one iris either for right or left. This limitation in critical IoT systems like ATMs decreases the system reliability against attacks, which we resolve in the proposed model. The performance of the proposed system is evaluated with an accuracy metric for the recognition rate over the used two data sets and outperformed the previous work.

## Proposed system

**Figure 1:**
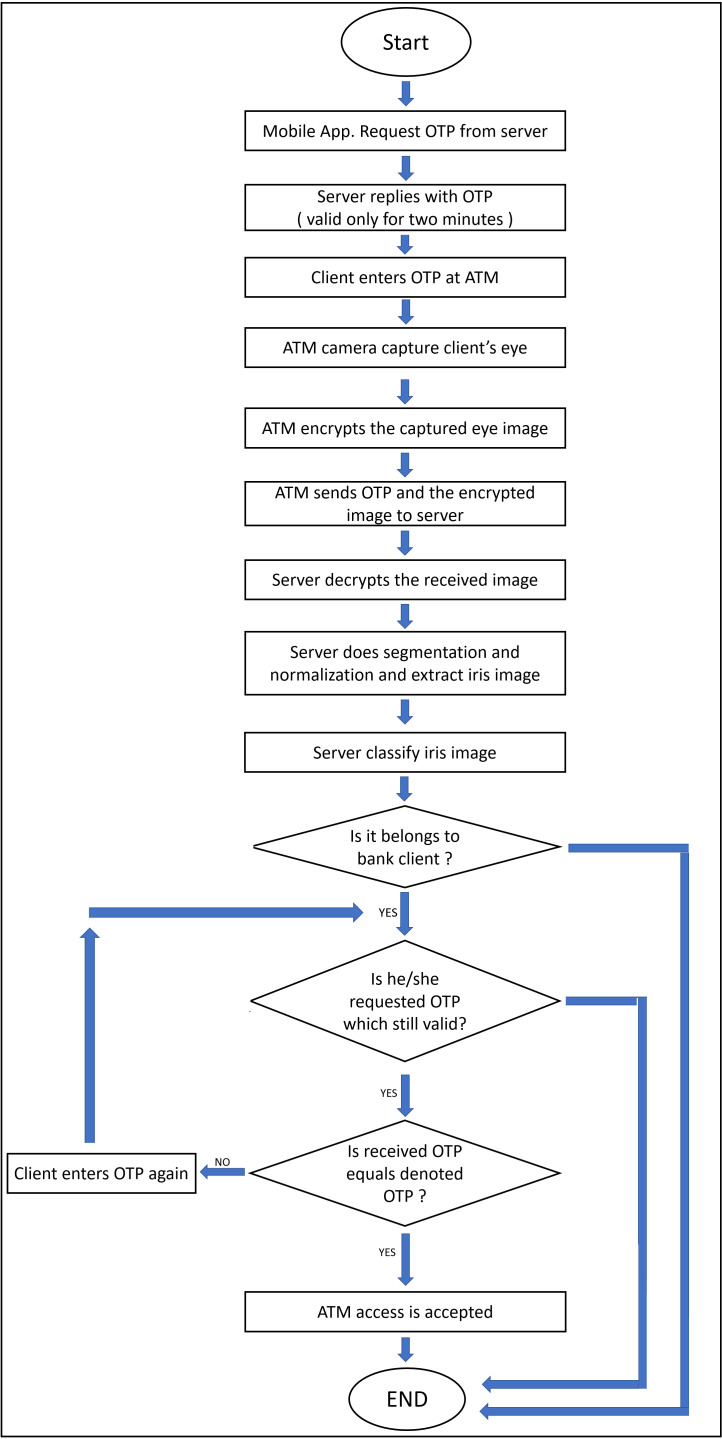
The flowchart of the proposed model.

The proposed system considers enrollment and authentication processes over the C-IoT authentication bank server. As shown in Fig. 1, the system starts by requesting a One-Time Password (OTP) by clients’ mobile applications from the bank’s server which replies with an OTP, that valid for two minutes. Then the client enters the acquired OTP to ATM which captures his/her eye images, encrypts them, and sends the OTP after the encryption of eye images to the bank’s server. Then the bank’s server decrypts the received eye images. Hence, it performs all the needed pre-processing operations—like segmentation and normalization—to extract the iris templates and classify them. It checks the correctness of received OTPs with the classified person, and finally sends the final decision about accessing ATMs to clients.

**Figure 2:**
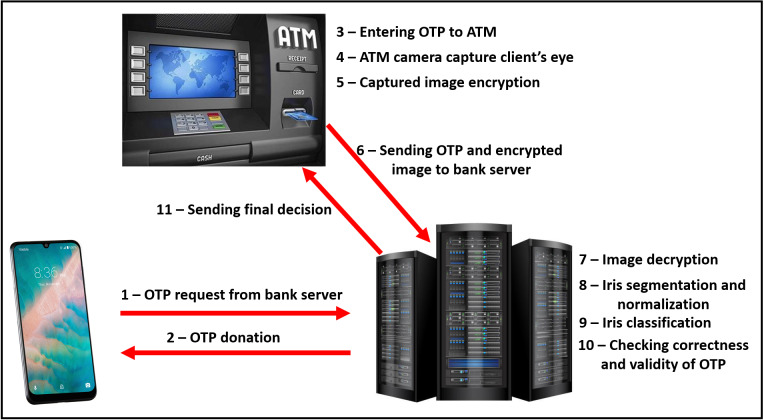
The explanatory sequence of the proposed model.

**Figure 3:**
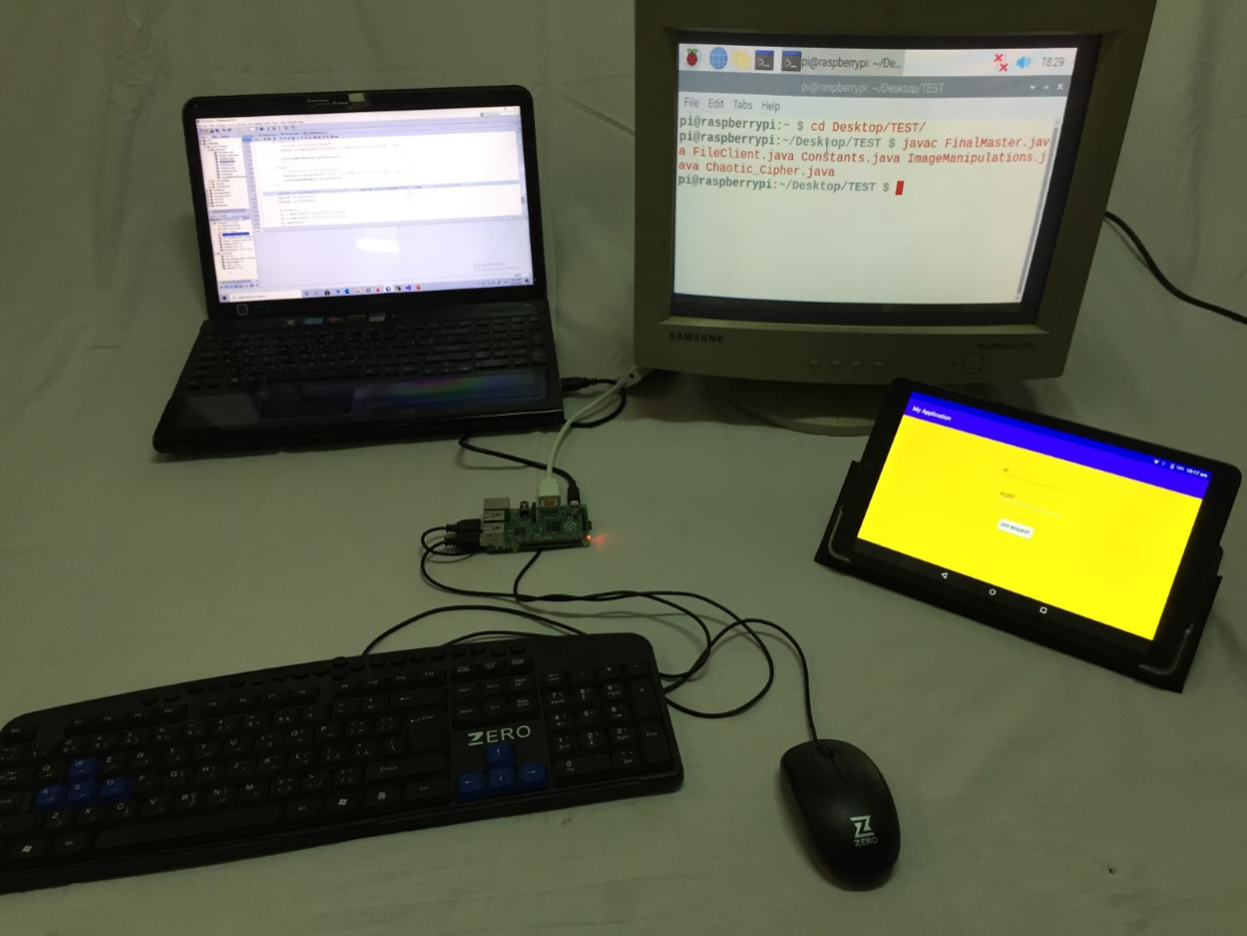
The practical physical connection and used devices in the experimental structure.

The proposed model consists of two main sides: (i) The client-side which is practically implemented by mobile phone, upon which the bank application exists, and raspberry pi-2 kit (Raspberry Pi Foundation, 2020), with other accessories like keyboard, mouse, and screen, which represent the ATM node. (ii) The server-side is practically implemented by a laptop. It is assumed that will be sensing devices to capture the iris images from model users. The physical connection and explanatory sequence diagram of the proposed model are shown in Figs. 2 and 3 respectively. The proposed model structure consists of the following key steps of the client-side: (i) Client’s Mobile Application requests One-Time Password (OTP) from the server, (ii) Client’s eye image acquisition by sensing devices of ATM, (iii) Client’s eye image encryption using a chaotic algorithm, and (iv) Finally, sending client’s encrypted eye image to the bank’s verification server over the internet.

**Figure 4:**
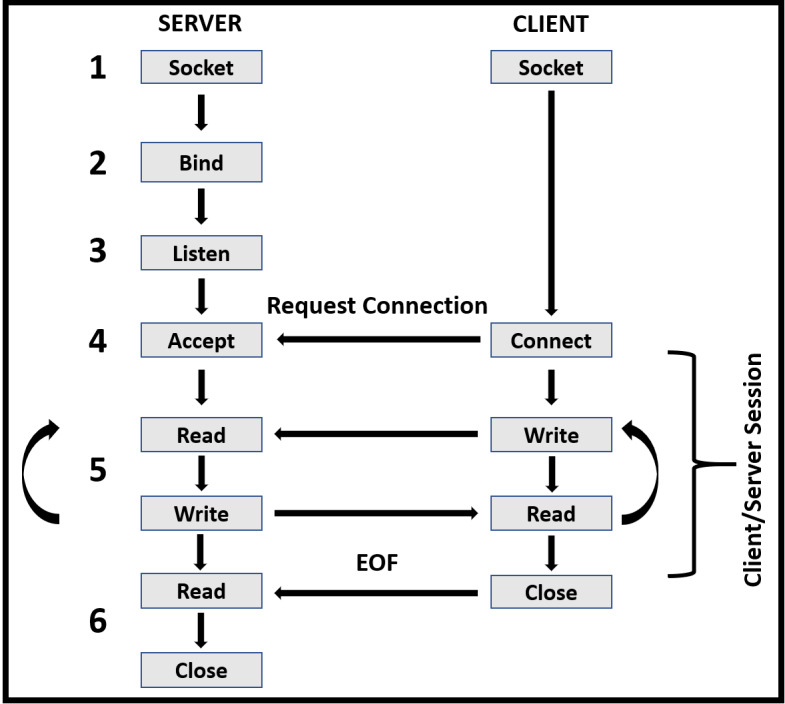
The classical client/server model is used by C-IoT proposed model.

The following key steps of the server-side: (i) Denoting OTPs to clients, (ii) Client’s encrypted eye image decryption, (iii) Iris segmentation and normalization, (iv) Extracting iris features using CNNs constructed model, (v) Client’s iris classification using FCNNs with Softmax layer, (vi) Checking the correctness of received OTP with the classified class OTP, (vii) Sending final decision about ATM accessing to clients. In the proposed system, the communication between the bank server and clients’ mobile applications or ATMs over the internet (Dizdarević et al., 2019) follows the classical client-server networking paradigm (Stevens et al., 2003) as shown in Fig. 4. The communication steps concerning the proposed environment are as follows:

1. The bank server creates a communication socket known as a listening socket.
2. Bank server binds its listening socket with any possessed IP address and specific port number which must be known to clients’ mobile applications and ATMs.
3. The bank server listens to any connection requests from clients.
4. The client’s mobile applications also create communication sockets and request connections to the bank server to receive the OTPs.
5. When the bank server receives a request from a client’s mobile application, it accepts the request and forks a new process that handles that client and denotes the required OTP to the client. Then the communication ends, and the forked process will be killed.
6. ATM also creates a communication socket, requests connection to the bank server, and sends the received OTP on mobile application and the eye image after encryption to the bank server.
7. When the bank server receives a request from an ATM, it accepts the request and forks a new process that handles it, performs decryption, segmentation, normalization, classification, and sends a final decision about accessing ATMs. Then the communication ends, and the forked process will be killed.
8. The bank server keeps listening to any coming communication requests from the client’s mobile applications or ATMs.

### Image encryption and decryption

The encryption is used to increase the proposed model’s security level while transmitting iris images over the internet which complicates the way of any system hackers (Stallings, 2016). Iris image encryption of the proposed model is done at the gray level. The implemented encryption algorithm is based on a chaotic key sequence generated by the sequence of the logistic map and sequence of states of Linear Feedback Shift Register (LFSR) as in Rohith, Bhat & Sharma (2014). It consists of two main steps

1. Encryption key sequence ($K\_Seq$) generation
2. Iris image encryption using the generated key sequence

To generate the encryption key sequence $K\_Seq$; two other non-negative integer finite sequences $K1$ and $K2$ of equal lengths, as shown in Eqs. (1) and (2), must be generated first.

(1) $$K\_Seq,\; K1,K2\; :\; \left\{ {1,2,3, \ldots \ldots ,p} \right\}\; \; \; \; \; \to \; \; \; \; \left[ {0,255} \right]$$

(2) $$p = w*l$$

where ($w$) and ($l$) are the width and the length of the iris image, respectively because the length of these sequences must equal the length of the iris image that will be encrypted. Terms of $K1$ sequence are generated first by the logistic map Eq. (3).

(3) $${X_{n + 1}} = r*\; {X_n}*\left( {1 - {X_n}} \right)$$

where $\left( r \right)$ is a parameter in the range of the closed interval $\left[ {2,4} \right]$ and ${X_{n + 1}}$ and ${X_n}$ are generic terms in the range of $\left[ {0,1} \right]$. With high values of $\left( r \right)$ like $\left( {r\; = \; 3.99} \right)$, ${K_1}$ will be a chaotic and unexpected sequence. then we round all terms of the obtained sequence by multiplying it by 255 to make sequence values in the range of gray level.

$K2\;$ sequence is generated by a sequence of states of an 8-bit Linear Feedback Shift Register. This sequence is defined inductively by the recurrence relation in Eq. (4) with an initial term $K{2_1}$, called the seed value, equals an integer in the range of [0,255], then perform XOR binary operation on bits of that seed, shift left it with the output of XOR operation and the result will be the second element of the $K2\;$ sequence and other sequence elements will be generated inductively in the same way.

(4) $$K{2_{n + 1}} = K{2_n} \gg \left( {\oplus K{2_n}} \right)\; \; \; \; \; \; \; \; \; \forall n\left( {1 \le n \le p - 1} \right)$$

where $\gg \left( x \right)$ denotes shift left operation with the value of $x$ bit, and $\oplus$ denotes the XORing operation of all bits of a term of a sequence.

Now $K\_Seq$ can be obtained directly from $K1$ and $K2$ sequences by XORing them as shown in Eq. (5)

(5) $$K\_Se{q_n} = \; K{1_n}\; (\oplus K{2_n})\; \; \; \; \; \; \; \; \; \; \; \; \forall n\left( {1 \le n \le p} \right)$$

Now, after obtaining encryption key sequence $K\_Seq$, encryption of iris image will be done by XORing each pixel of an iris image with its corresponding element in the key sequence as shown in Eq. (6).

(6) $$IM\_ENC\left( {x,y} \right) = \; K\_Se{q_{x + \left( {y - 1} \right)*w}}\oplus IM\_ORIG\left( {x,y} \right)\; \; :\forall x,y\left( {\left( {1 \le x \le w} \right) \wedge \left( {1 \le y \le l} \right)} \right)$$

where $IM\_ORIG\left( {x,y} \right)$ and $IM\_EN{C {\left( {x,y} \right)}}$ denotes the value of the original and encrypted image pixels at $\left( {x,y} \right)$.

With respect to key management which to the management of cryptographic keys in a cryptosystem where these keys must be chosen carefully and distributed and stored securely. In general, the distributed key management scheme can be further divided into symmetric schemes and public-key schemes. The symmetric-key method uses a secret key which is known as an asymmetric key the same key is used for a cryptographic system. The second method named Asymmetric key that uses two related keys (i.e., a public key and a private key) where the public key may be known by anyone; the private key should be under the sole control of the entity that owns the key pair. In the proposed system, the server and ATM generated the key in the same way for verification purposes at each side, exclusively. Therefore, in this suggested system, there is no need for the exchange process between them due to the idea of this work principally focuses on building an efficient iris recognition system and not predominantly on key management. Furthermore, for the effectiveness of this system will be a need for a security method for the secure exchange of this symmetric key especially with the increasing number of nodes belongs to the banking system. For key management that can be suitable for this work, it can be considered that used the symmetric key scheme, which is based on private key cryptography, whereby shared secrets can be used to authenticate legitimated machines (in our system are the server and ATM) to provide secure communication between them over the IoT system. This shared secret key can be distributed via secure channels and it is the same key for both entities of the banking system.

The decryption operation is typically the reverse order of these steps of encryption. As shown in Rohith, Bhat & Sharma (2014), this LFSR methodology provides cryptographically better results as compared to the methods that encrypt using a logistic map scheme alone, it provides a high degree of secrecy and security. The original iris image and the encrypted image are highly uncorrelated and perceptually different. For the above reasons, this version of chaotic encryption is incorporated in the proposed model; to add secure iris transmission between ATMs and the bank server over the internet.

### Iris segmentation and normalization

The segmentation step, as the more critical in the recognition operation, is proposed. The generated iris template by Masking Technique (MT) (Gad et al., 2016, 2018) is stored in the IoT server. The decrypted iris image A (x, y) is the original iris image in this stage. The simplest operation, to remove the upper and lower parts of the iris, occluded by eyelashes and eyelids, is by changing all the pixels above and below the vertical diameter of the pupil to zero value. Let iris image $I\left( {x,y} \right)$ has $m*n$ pixels, ${\rm \; }\forall {\rm \; }1 \le y \le n$, the eyelashes/eyelids removing mask [${M_e}\left( {x,y} \right)$] identified as:

(7) $${M_e}\left( {x,y} \right) = \left\{ {\matrix{ {\hskip-11pt}{0\; :1 \le x \le Index\left( {{P_4}\left( {x,y} \right)} \right),Index\left( {{P_2}\left( {x,y} \right)} \right) \le x \le m} \cr {1\; \; \; \; \; \; \; \; \; \; \; \; \; \; \; \; \; \; \; \; \; :Index\left( {{P_4}\left( {x,y} \right)} \right) < x < Index\left( {{P_2}\left( {x,y} \right)} \right),\; } \cr } } \right.$$

**Figure 5:**
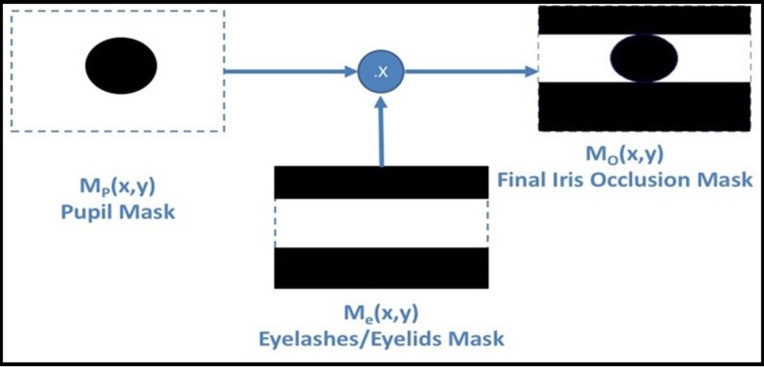
MT mask generation process.

The final mask [${M_o}\left( {x,y} \right)$], is shown in Fig. 5, identified in binary format as:

(8) $${M_o}\left( {x,y} \right)\; = \; {M_p}\left( {x,y} \right)\; \times \; {M_e}\left( {x,y} \right)$$

A fixed template size (such as 60 × 90 *pixels*) is generated. This was unsuitable for some images in datasets, due to image size and resolution. Some modifications were done over MT; the (*N*) pixels to the left and right of the localized pupil are concatenated. Iris template is created by mapping the selected pixels on a fixed size (60 × 2*N*) matrix.

**Figure 6:**
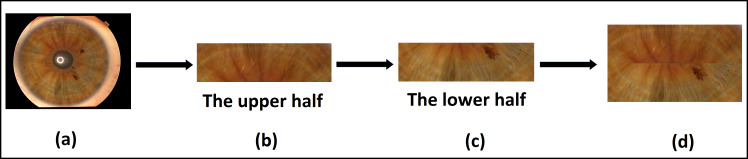
The proposed template generation for the Phoenix dataset. (A) Original image, (B) the upper half of iris, (C) the lower half of iris, (D) the final iris template.

In the case of the Phoenix dataset, the dataset is available in a segmented state as shown in Fig. 6A. The upper half and the lower half of iris images were selected, each of dimensions of (350*100) pixels, then concatenated together, as shown in Figs. 6B, 6C, and 6D. We use the final concatenation parts of the image by dimensions of (350*200) as an iris template in the next stage of feature extraction. This solution does not consider the rotation of both the camera and the eye. Moreover, a bit of iris information is lost in the left and right collarette zones.

### Deep CNN-based feature extraction and classification

**Figure 7:**
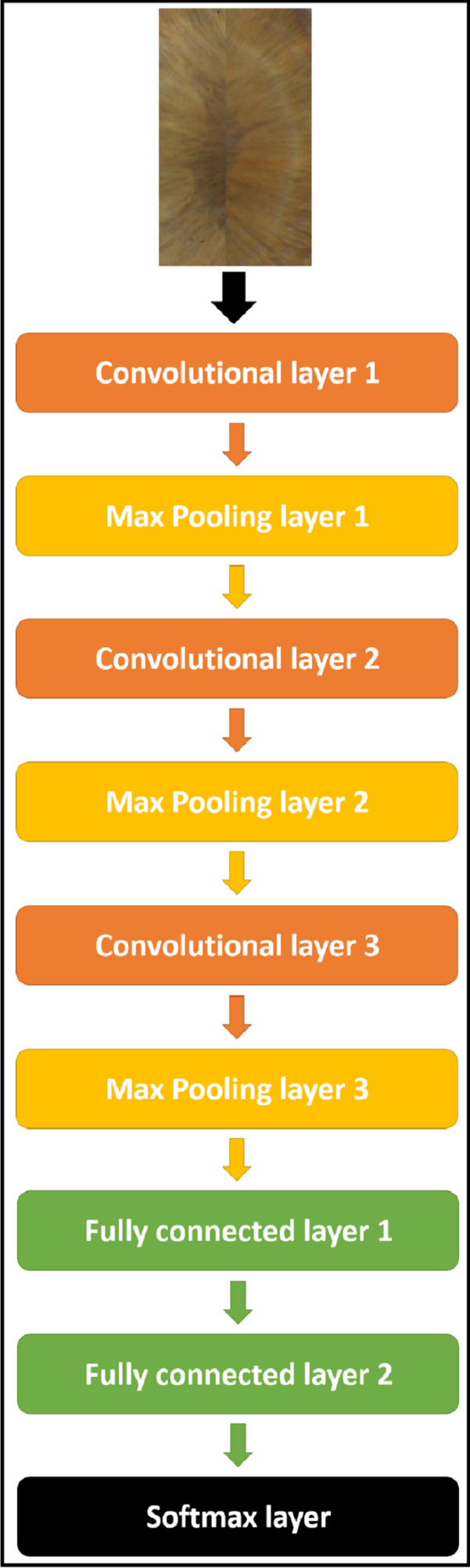
The proposed CNN model structure for all datasets.

Different CNNs with different architectures (as it will be shown in experimental results), were used to extract the deep features from iris images for the dual iris in the case of Phoenix and the lonely iris in the case of UBIRIS.V1. The target is to find an architecture that gives the highest recognition rate. After a lot of experiments, we gain a complete model for all datasets consists of CNN as an iris feature extractor, which consists of “3” convolutional layers, “3” max-pooling layers, “3” RELU activation layers (Aggarwal, 2018), and FCNN of “2” fully connected layers, and “1” SoftMax layer (Courville, Goodfellow & Bengio, 2016) as a classifier. The specific configuration of the overall network is illustrated in Table 1 and Fig. 7.

**Table 1:** The proposed CNN architecture and configuration for both datasets.

Layer name	No of filters	Filter size	Stride size	Padding
Conv1	100	3*3	1*1	Valid
RELU	n/a	n/a	n/a	n/a
Max pooling	1	2*2	2*2	Valid
Conv2	150	3*3	1*1	Valid
RELU	n/a	n/a	n/a	n/a
Max pooling	1	2*2	2*2	Valid
Conv3	200	3*3	1*1	Valid
RELU	n/a	n/a	n/a	n/a
Max pooling	1	2*2	2*2	Valid
Fully connected layer1	250 nodes	n/a	n/a	n/a
Fully connected layer2	150 nodes	n/a	n/a	n/a
Softmax layer	n/a	n/a	n/a	n/a

### System reliability

**Figure 8:**
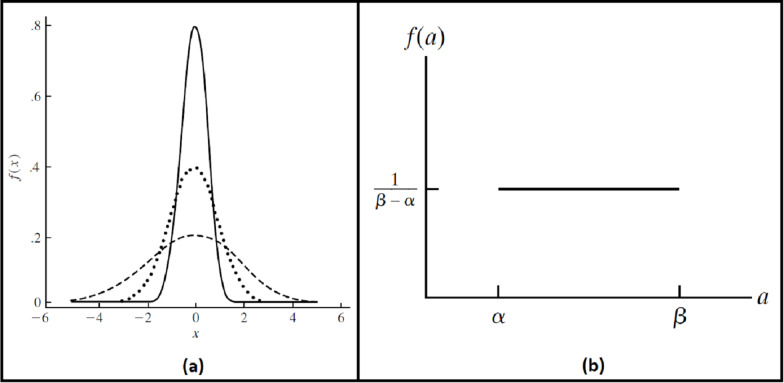
The probability density functions. (A) Gaussian (Normal) distribution. (B) Uniform distribution.

To ensure the reliability and robustness of the proposed system, we explored how it deals with noised data and how the accuracy of the recognition rate of the proposed model would be affected. It is assumed that image acquisition at ATMs may gain some external noise due to several reasons like system attacks, environmental dust, interference noise on iris sensing devices, or bad iris acquisition due to faults of system users, different illumination states…etc. Two different kinds of noise are added. The first kind is generated randomly from a Gaussian distribution, with a mean (${\rm \mu}$) zero and several standard deviations (${\rm \sigma}$) (Rice, 2007). The other type of noise is generated by uniform distribution at different intervals (Ross, 2014). Probability density functions for these distributions (Wackerly, Mendenhall & Scheaffer, 2008) are shown in Fig. 8; Eqs. (9), and (10). These randomly generated noise values are added per pixel of iris images.

(9) $$f\left( x \right) = \; \displaystyle{1 \over {{\rm \sigma} \sqrt {2{\rm \pi}} }}*\; {e^{\textstyle{{ - {{\left( {x - {\rm \mu}} \right)}^2}} \over {2*{{\rm \sigma}^2}}}}}\; \; \; \; \; \; \; \; \; \; \; \; \; \; - \infty < x < \; + \infty$$

(10) $$f\left( x \right) = \; \left\{ {\matrix{ {\displaystyle{1 \over {\rm {\beta - \alpha }}}\; \; \; \; \; \; \; \; \; \; \; \; \; \; \; \; \; \rm \alpha \lt {\it x} \lt  {\rm \beta}} \cr {\; \; \; \; \; \; 0\; \; \; \; \; \; \; \; \; \; \; \; \; \; \; \; \; \; \; \; \; \; \; \rm otherwise\; \; } \cr } } \right.$$

**Figure 9:**
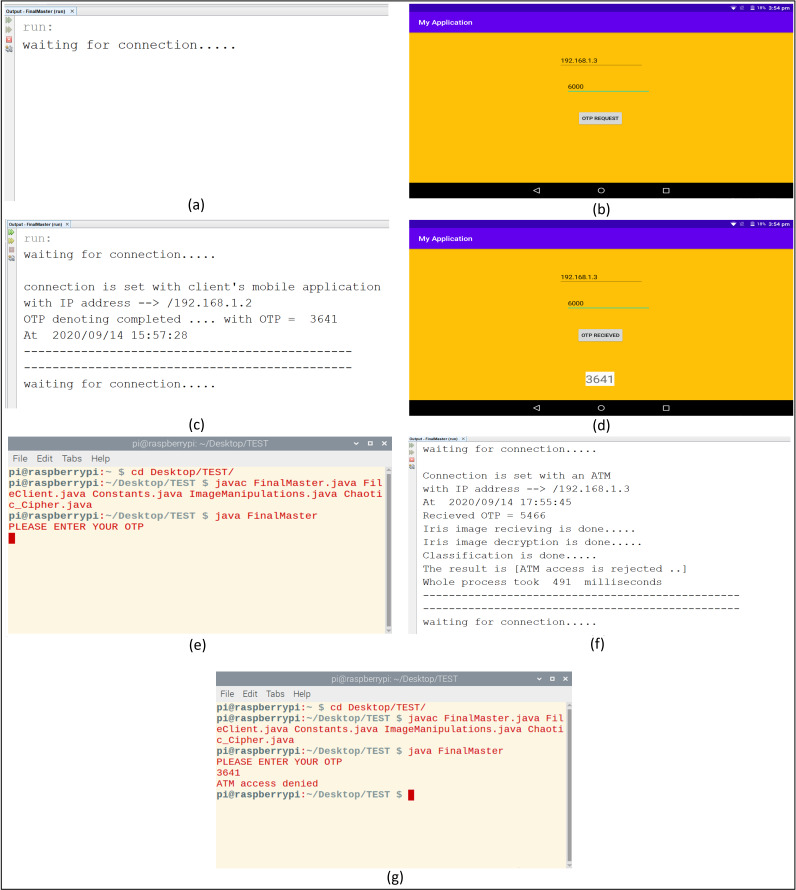
The experimental connection action sequence of the proposed system. (A) Bank server is waiting for connections. (B) Client requests an OTP. (C) OTP donation by bank server. (D) The client receives an OTP. (E) ATM enters received OTP and captures eye image. (F) The bank server determines the final decision. (G) The client receives the final decision about accessing the ATM.

The overall practical steps of the experiments will be viewed before dealing with the experimental results of each step alone. First, the bank server task is set up for listening to connection requests from clients’ mobile applications or ATMs. When the client’s mobile application requests an OTP, the bank server accepts the request and denotes an OTP to the client. When ATM’s client request ATM access by entering the received OTP and capturing his/her eye image, the server receives his/her request, perform all needed operations like segmentation, normalization, classification, and OTP correctness checking. Then the final access decision is sent to clients by accepting or rejecting ATMs access. These sequences of actions at mobile applications, ATMs, and the bank server are shown in Fig. 9.

## Experimental results

### Iris acquisition

Two publicly available datasets were used in the experiments which capture the iris eyes using visible light cameras; so, they are suitable for ATMs, which are the target environments of the proposed system. These datasets are Phoenix and UBIRIS.V1. The later dataset incorporates images with several noise factors, thus permitting the evaluation of more robust iris recognition methods. The iris images in these datasets are captured under different situations of pupil dilation, eyelids/eyelashes occlusion, the slight shadow of eyelids, specular reflection, etc.

The iris images of the Phoenix dataset have a resolution of 576 × 768 pixels. The images were taken by TOPCON TRC50IA optical device connected to SONY DXC-950P 3CCD camera. Also, all iris images of the UBIRIS dataset are 8-bit Gray-level of (. JPEG) file format. Its iris is imaged with a resolution of 200 × 150 pixels. The irises were taken by Nikon E5700 camera with a Focal Length of 71 mm and Exposure Time of 1/30 s. The third dataset used in our system; to strengthen our model, is the CASIA V4-interval dataset. CASIA V4-interval dataset is the latest dataset captured by CASIA self-developed close-up iris camera, all iris images are 8-bit Gray-level (. JPEG) files, collected under near-infrared (NIR) illumination. Its iris images with resolution (320*280) pixel. The summary of the configuration of these datasets is shown in Table 2.

**Table 2:** Description summary of the used two datasets.

	Visible light vision datasets		*Near-Infrared (NIR)* datasets
	Phoenix	UBIRIS. V1	*CASIA V4 interval*
Description	384 iris Images taken from 64 subjects	1,877 images collected from 241 subjects	2,641 iris image taken from 249 subjects
Images resolution	576*768 pixels	200*150 pixels	320*280 pixels
Image format	(.PNG)	(.JPEG) (Check)	JPEG
Subjects used in localization	ALL	ALL	ALL
Subjects used in classification	60	50	55
Samples per subject	3 right and 3 left	5	not regular

In our experiments, we used a Sony laptop with Intel CORE i5 CPU and RAM of 6 GB, a raspberry pi 2 kit, and ALCATEL one touch Pixi3 tablet with an Android operating system of 5.1 version. They were used as the server-side, ATM node, and the client mobile phone respectively. All these components were shown previously (in Figs. 2 and 3). Java, Python, and MATLAB programing languages were used for experiments. PyCharm, NetBeans, MATLAB, and Android studio are used as integrated development environments in overall experiments.

### Iris encryption and decryption

In all experiments on light vision dataset images, the values of the parameters needed to generate $K\_Seq$, $K1,$ and $K2$ sequences are chosen as shown in assignments Eq. (11).

(11) $$r = 3.99\; \; \; \; \; \; \; \; \; \; \; \; \; \; \; \; \; \; \; \; \; \; \; \; \; \; {X_1} = \; 0.1\; \; \; \; \; \; \; \; \; \; \; \; \; \; \; \; \; \; \; \; \; \; \; \; \; \; K{2_1} = {\left( {0100101} \right)_2}$$

**Figure 10:**
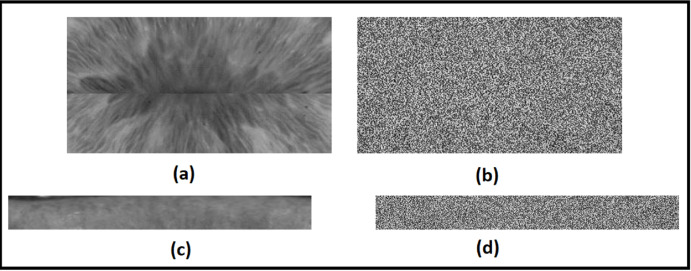
Results of Chaotic encryption and decryption. (A) Original image from the phoenix dataset. (B) The encrypted image of the original. (C) Original image from the UBIRIS dataset. (D) The encrypted image of the original.

Figure 10 shows the results of the chaotic encryption algorithm on light vision datasets.

### Iris segmentation and normalization

In MT, the mask size (*N*) controls the iris region. In Table 3, the accuracy changed according to the value of the (*N*) parameter. The specular reflect in the pupil is one circular white spot; our algorithm hardly detects a pupil with such environmental nature. The range of (30–35) for mask size (*N*) in MT achieves the best accuracy. When $(N < 30)$ the final iris mask expanded, including sclera pixels gradually. For $\; (N > 35)$, the mask loses more information from the iris circle.

**Table 3:** The segmentation success rate for every *N* pixels in MT.

Mask size (*N*) (pixel)	Success rate (%)
60	78.268
55	79.542
50	80.815
45	82.089
40	87.882
35	93.674
30	99.467

### Training and classification using CNNs

Many trials of experiments were done to arrive at the best tuning of the network parameters of the proposed model architecture previously shown in Table 1. Some metrics were measured; to evaluate the performance of the proposed model. The first metric is the recognition accuracy rate (${{\rm A}_{RR}}$%) which is the portion of correctly iris classifications to the total number of classified irises declared in Eq. (12). The other metrics are precision, recall, F1-Score, and the training time of the proposed CNN model.

(12) $${{\rm A}_{RR}}{\rm \; }\left( {\rm \% } \right) = {\rm \; }\displaystyle{{{\boldsymbol{N}_{\boldsymbol{c}}}} \over {{\boldsymbol{T}_{\boldsymbol{c}}}}}{*}100$$

where (***N_c_***) represents the number of correct iris classifications and (***T_c_***) represents the total number of classified irises. We divided each data set into two subsets, the first subset is used for training CNN to get the best tuning of the parameters, and the second is used for testing each CNN configuration to get its ${{\rm A}_{RR}}$. In all datasets the ratio between training to testing datasets ($R_{\rm test}^{\rm train}$) is shown in Eq. (13).

(13) $$R_{\rm Test}^{\rm Train} = \; \displaystyle{{\rm \left| {Train} \right|} \over {\rm \left| {Test} \right|}} = 4:1$$

where $\left| {\rm Train} \right|$ and $\left| {\rm Test} \right|$ are the cardinality of training and testing subsets, respectively.

During experiments, different CNN architectures differ in their configuration of convolutional layers, max-pooling layers, RELU layers, kernel sizes, strides, and their fully connected layers. Different training parameters (Vinayakumar et al., 2020) like learning rate, number of epochs, patch sizes, and dimensions of input iris images were used. Tables 4–7 show a part of the experiments done on the model until reaching the best architecture that gives the highest recognition rate. The best architecture for all datasets together, is shown in Table 6 and its configuration was previously described in Table 1. We did not any experiments on UBIRIS. V1 or CASIA V4 with dimensions of (128 * 128) as shown in Table 7 because the dimensions of the iris template are less than this, so we avoided generating extra features and depending on them.

**Table 4:** Accuracy of recognition rates obtained for different CNN architectures using the input image size of (256 × 64) pixels.

Configuration	Phoenix		CASIA V4		UBIRIS
	Left	Right	Right	Left	
[20 80 120]*	93.33	90	96.96	96.96	94
[5 50 100]	93.33	8166	93.93	93.93	96
[5 50 120]	91.66	90	98.48	95.45	94
[5 40 120]	90	88.33	89.39	86.36	92
[120 120 120]	85	88.33	98.48	96.96	96
[20 70 160]	91.66	90	95.45	92.42	90
[120 100 80]	88.33	86.66	93.93	87.87	96
[120 80 50]	90	90	100	95.45	96
[20 80 140 256]	81.66	88.33	80.30	83.33	92
[5 50 100 150]	85	93.33	83.33	84.84	90
[10 80 120 180]	91.66	93.33	92.42	86.36	94
[20 70 160 200]	88.33	78.33	89.39	87.87	94

**Note:**

* Where in the pattern of [×1 ×2 ×3]: ×1, ×2, and ×3 indicates the number of kernels in each convolutional layer.

**Table 5:** Accuracy of recognition rates obtained for different CNN architectures using the input image size of (128 × 64) pixels.

Configuration	Phoenix		CASIA V4		UBIRIS
	Left	Right	Right	Left	
[6 50 150]	93.33	98.33	95.45	93.93	94
[100 150 200]	91.66	90	98.48	90.90	92
[10 50 250]	95	91.66	95.45	93.93	94
[10 100 200]	93.33	88.33	96.96	90.90	96
[120 120 120]	96.66	85	95.45	92.42	94
[100 200 300]	93.33	86.66	95.45	90.90	90
[20 70 160]	96.66	98.33	98.48	93.93	96
[120 80 50]	93.33	93.33	93.93	93.93	96
[10 40 80]	96.66	93.33	93.93	92.42	92
[10 50 100 200]	90.00	91.66	92.42	90.90	94
[50 100 150 250]	90.00	90	89.39	87.87	94
[100 150 200 250]	93.33	91.66	92.42	92.42	92

**Table 6:** Accuracy of recognition rates obtained for different CNN architectures using the input image size of (64 × 64) pixels.

Configuration	Phoenix		CASIA V4		UBIRIS
	Left	Right	Right	Left	
[10 50 100]	95	96.66	98.48	98.48	98
[10 40 80]	98.33	98.33	96.96	98.48	96
[10 100 150]	98.33	95	95.45	96.96	92
[20 70 160]	96.33	95	98.48	95.45	96
[120 120 120]	95	95	96.96	98.48	94
[100 150 200]	100	100	98.48	100	98
[20 60 120 180]	90	96.66	92.42	95.45	96
[10 50 100 150]	93.33	95	95.45	92.42	92
[50 100 150 200]	95	96.66	96.96	93.93	94
[100 150 200 250]	95	95	95.45	92.42	98
[10 50 100]	95	96.66	98.48	98.48	96
[10 40 80]	98.33	98.33	96.96	98.48	94

**Table 7:** Accuracy of recognition rates obtained for different CNN architectures using the input image size of (128 × 128) pixels.

Configuration	Phoenix left eye	Phoenix right eye
[20 80 120]	83.33	90
[5 50 100]	95	93.33
[5 50 120]	86.66	91.66
[5 40 120]	88.33	96.66
[120 80 50]	93.33	88.33
[5 50 100 150]	93.33	88.33
[10 80 120 180]	95	90
[20 70 160 200]	90	90

**Figure 11:**
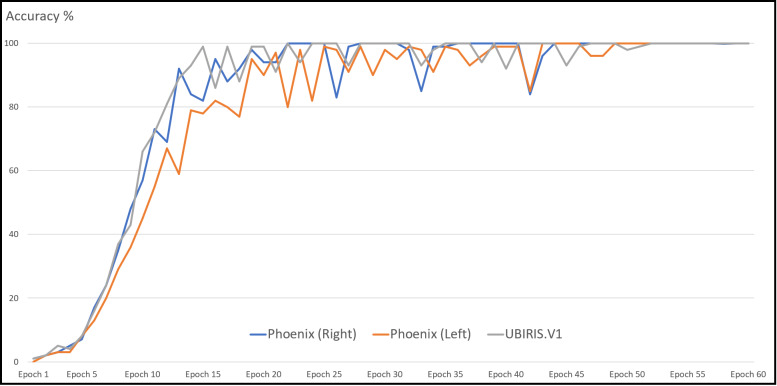
Average accuracy curve for the training of datasets.

**Figure 12:**
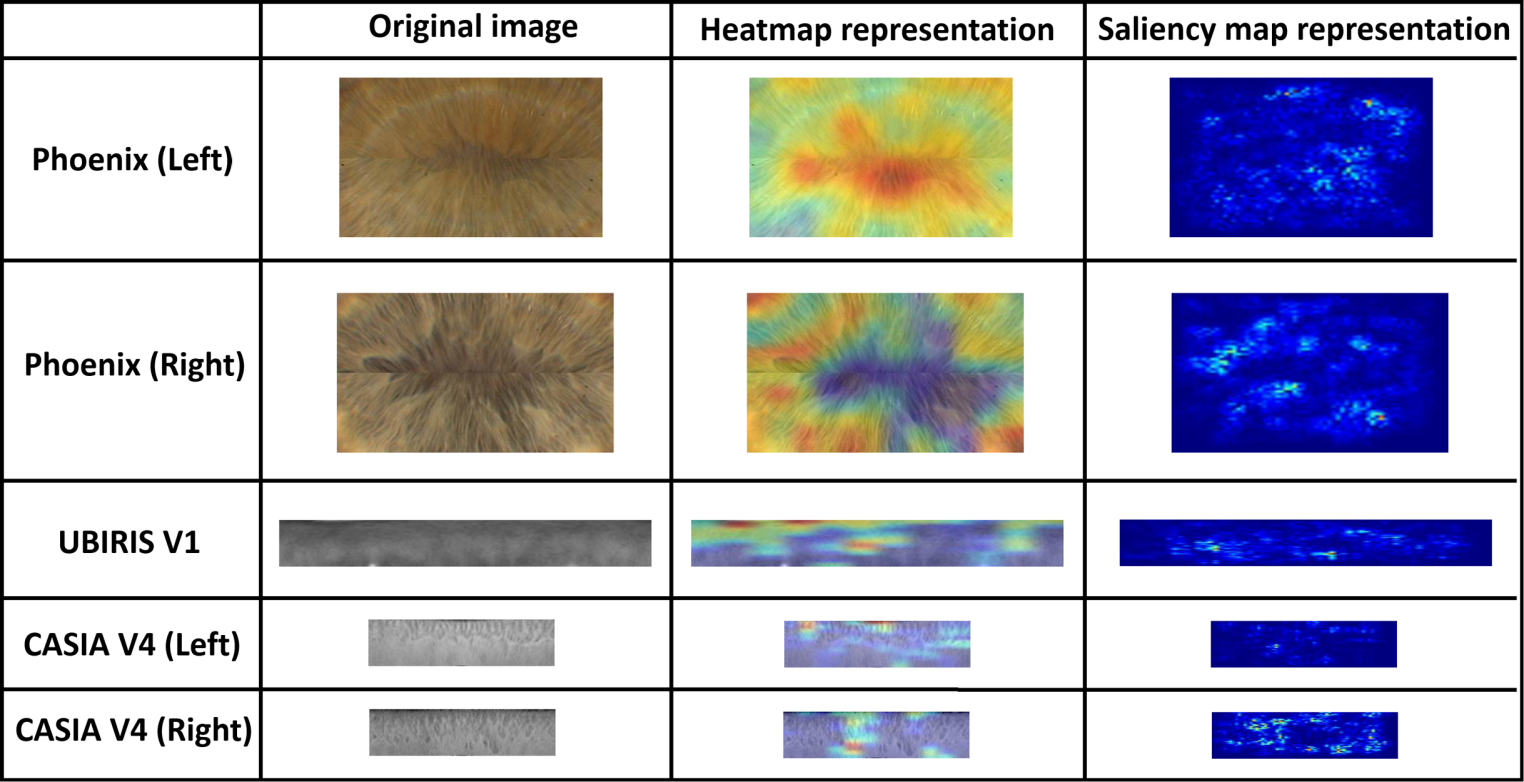
Heatmap representation of activation functions and saliency map representation for all used datasets.

With the Phoenix dataset, the model was trained with 120 iris images for 60 classes of data in the case of each iris left and right and tested against 60 iris images. The model correctly classified all iris images for both left and right irises with an accuracy of recognition rate of 100% for left and right iris. With the UBIRIS. V1 dataset the model was trained with 200 iris images for 50 classes of data and tested against 50 iris images. The model correctly classified all iris images except only one with an accuracy of recognition rate of 98%. With the CASIA V4 interval dataset, we trained our model with 265 iris images for 55 classes of data in the case of each iris left and right and tested it against 66 iris images. The model correctly classified 65 iris images with the left eye and 66 images with the right eye. The accuracy of the recognition rate of 98.48% and 100% for the left and right iris, respectively. So, the model has an accuracy of 99.24% of the overall CASIA V4-interval dataset. So, the overall accuracy of the proposed model is 99.33% as shown in Table 6. Figure 11 shows the average accuracy curves for training for visible light vision datasets. Figure 12 shows heatmap representation of activation functions and saliency map representation for all used datasets.

Because our problem is a multi-class classification problem, so we have a Precision, recall, and F1-Score measure for each class of the clients of the system as shown in Eqs. (14), (15) and (16).

(14) $$\rm Precision\; \left( {Class = \; {\it x}} \right) = \; \; \displaystyle{{TP\left( {class = {\it x}} \right)} \over {TP\left( {class = {\it x}} \right) + FP\left( {class = {\it x}} \right)}}$$

(15) $${\rm Recall}\; \left( {{\rm Class} = \; {\it x}} \right) = \; \; \displaystyle{{{\rm TP}\left( {{\rm class} = {\it x}} \right)} \over {{\rm TP}\left( {{\rm class} = {\it x}} \right) + {\rm FN}\left( {{\rm class} = {\it x}} \right)}}$$

(16) $$\rm F1 - Score\; \left( {Class = \; {\it x}} \right) = \; \; 2*\displaystyle{{Precision\left( {class = {\it x}} \right)*Recall\left( {class = {\it x}} \right)} \over {Precision\left( {class = {\it x}} \right) + Recall\left( {class = {\it x}} \right)}}$$

where TP, FP, FN, and $x$ stands for true positive, false positive, false negative, and the number of client’s class, respectively.

All of the 60 classes of the Phoenix dataset, for left and right iris, have 1, 0.0163, and 0.032 for precision, recall, and F1-Score, respectively. With the UBIRIS. V1 dataset, 49 class has 1, 0.02, and 0.039 for precision, recall, and F1-Score, respectively.

Concerning the time sequence of operations between the bank server and ATMs, this time includes receiving encrypted images, performing iris preprocessing operations of segmentation and normalization, passing it to the classifier, and replying to ATMs with the final decision, in our experiments this time is within the average of (0.5 s), which considered a relatively small period as shown previously in Fig. 9. To deal with the lack of dataset images per subject during training, a simple data augmentation operation was done. This augmentation was based on simple image processing operations on iris images like concatenating some parts from the top, bottom, left, or right of iris images. And these new images were added as a part of the training set.

With each of the experiments in Tables 4, 5, 6, and 7, the number of epochs, batch size, and learning rate was varying until reaching the best values. It is found that a suitable number of epochs needed for training which gives us the highest recognition rate was 60 epochs with a batch size of 40, a learning rate of 0.001 with categorical cross-entropy loss function.

Concerning training time, with the final configuration of the CNN model, the training time of the Phoenix dataset was about 17 min for each right and left sub-sets, 22 min for UBIRIS. V1 dataset and the training time of the CASIA V4 interval dataset was about 25 min for each right and left iris sub-sets. It is considered relatively low training time comparing with others, like (Al-Waisy et al., 2018), who addressed the use of deep learning in iris recognition with training time exceeds 6 h. Table 8 shows that the proposed CNNs model has competitive results compared to state-of-the-art methods in terms of recognition accuracy.

**Table 8:** Comparison of the proposed CNN model with other works in terms of accuracy of the recognition rate.

Approach	Dataset	Feature extraction	Classification	Recognition accuracy %
Singh et al. (2020)	UBIRIS.V2	Integer Wavelet Transform (IWT)	Normalized Hamming distance	98.9
Khanam et al. (2019)	CASIA-Iris-V1	Haar wavelet and Daubechies wavelet	feedforward neural network	94.76
Lozej et al. (2019)	CASIA Thousand	Pre-trained Xception	Pre-trained DeepLabV3+ with MobileNet	97.46
Rana et al. (2019)	CASIA-Iris-V4	PCA and DWT	SVM	95.40
Alaslani & Elrefaei (2018)	CASIA-Iris-Interval	pre-trained Alex-Net model	Multi-Class SVM	89
Al-Waisy et al. (2018)	CASIA-Iris-V3	Convolutional Neural Network	Softmax classifier + fusion	100
Minaee, Abdolrashidiy & Wang (2016)	CASIA-Iris-Thousand	pre-trained VGG-Net	Multi-Class SVM	90
Saminathan, Chakravarthy & Chithra Devi (2015)	CASIA-Iris-V3-interval	Intensity image	Least square method of quadratic SVM	98.50
Dhage et al. (2015)	Phoenix	Discrete Wavelet Transform (DWT) and Discrete Cosine Transform (DCT)	Euclidean distance	88.50
Bharath et al. (2014)	CASIA-Iris-V3	Radon transform and gradient-based isolation	Euclidean distance	84.17
Sundaram & Dhara (2011)	UBIRIS. V1	Haralick features	Probabilistic Neural Networks (PNNs)	97
The proposed model	CASIA-Iris-V4	Convolutional Neural Network	Softmax classifier	99.24
	UBIRIS.V1			100
	Phoenix			98

### Testing the effect of noise on the proposed model

**Figure 13:**
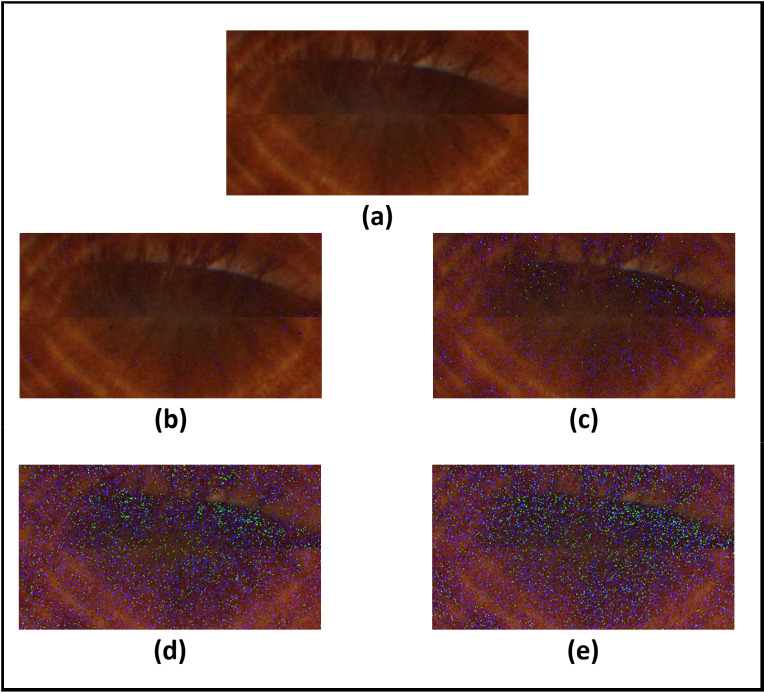
Results of adding gaussian noise with different standard deviations. (A) Original image. (B) Standard deviation = 5. (C) Standard deviation = 10. (D) Standard deviation = 15. (E) Standard deviation = 20.

**Figure 14:**
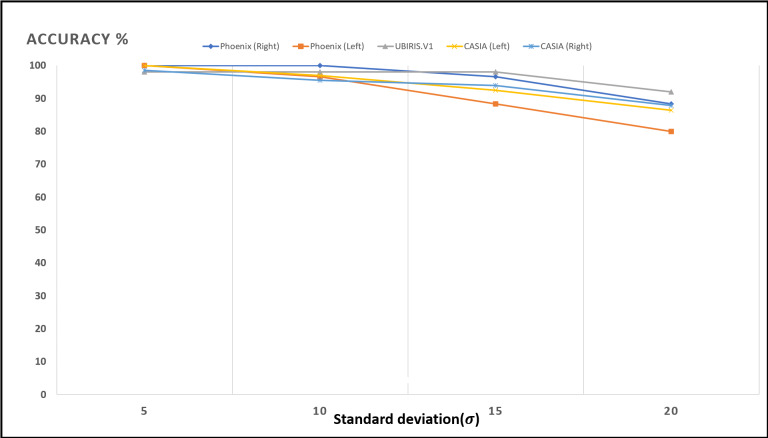
Accuracy of recognition degradation curve after adding Gaussian noise with different standard deviations.

**Figure 15:**
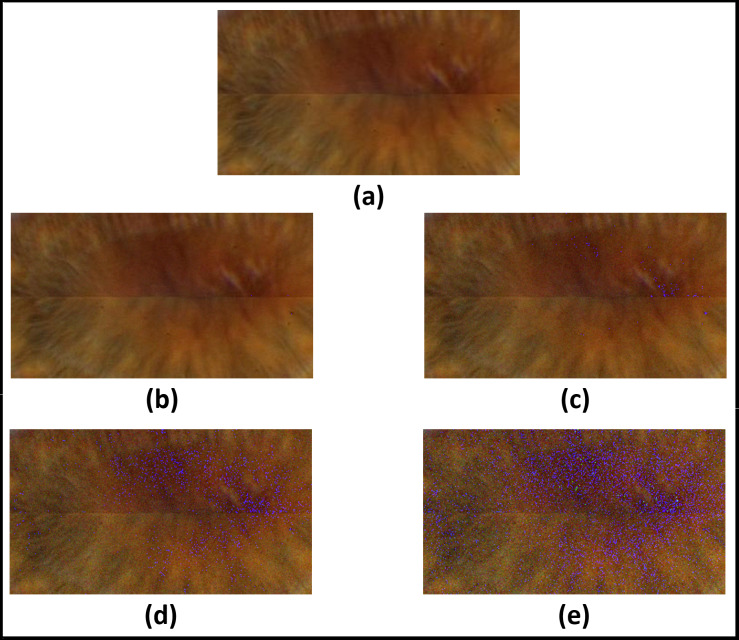
Results of adding uniform noise within different intervals. (A) Original image. (B) Within interval of [−5, +5]. (C) within interval of [−10, +10]. (D) within interval of [−15, +15]. (E) within interval of [−20, +20].

**Figure 16:**
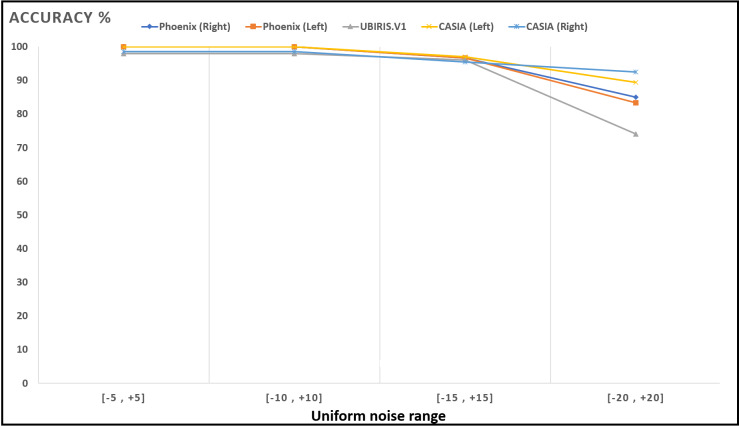
Accuracy of recognition degradation curve after adding uniform noise at different intervals.

Figures 13–16; Tables 9 and 10 show the obtained results with Phoenix, UBIRIS. V1 and CASIA V4 datasets for the added noise from Gaussian distribution and the uniform distribution (Walpole et al., 2012), respectively. These results are based on our final CNN configuration that yields the highest recognition rate in the ideal case of iris images without any added noise.

**Table 9:** The model recognition rate against noised iris images (Gaussian noise).

Standard deviation	UBIRIS	Phoenix		CASIA V4	
		Left iris	Right iris	Left iris	Right iris
5	98	100	100	100	98.484
10	98	96.666	100	96.969	95.454
15	98	88.333	96.666	92.424	93.939
20	92	80.000	88.333	86.363	87.878

**Table 10:** The model recognition rate against noised iris images (uniform noise).

Interval	UBIRIS	Phoenix		CASIA V4	
		Left iris	Right iris	Left iris	Right iris
$\left[ { - 5, + 5} \right]$	98	100	100	100	98.484
$\left[ { - 10, + 10} \right]$	98	100	100	100	98.484
$\left[ { - 15, + 15} \right]$	96	96.666	96.666	96.969	95.454
$\left[ { - 20, + 20} \right]$	74	83.333	85.000	89.393	92.424

These results show how well the proposed model deals with noised iris images from different noise distributions like Normal and uniform distributions. The proposed system shows a low degradation of recognition accuracy rates in the case of using noised iris images.

## Discussion

The proposed iris recognition system for ATMs verification shows good results concerning the accuracy of recognition rate and training time for Phoenix, UBIRIS. V1 and CASIA V4 datasets. The system has a low degradation of recognition accuracy rates in the case of using noisy iris images with different noise distributions like normal and uniform distributions. It also provides a secure method of iris template transmission over the communication channel between ATMs and the bank servers by protecting the iris using chaotic encryption. We need to increase the number of classes of clients used for training our system; to test its scalability over large communities of clients in real-life scenarios. In future work, we planned to extend this work by using different types of encryption algorithms as well as focusing on applying different key management schemes for secure the distribution of the key among different entities for IoT-based applications in diverse areas.

## Conclusion

In this article, an efficient full verification system for ATMs, based on mobile applications is proposed. Iris recognition using deep Convolutional Neural Network (CNN) as a feature extractor and fully connected neural network (FCNN), with the Softmax layer as a classifier, is presented. A bank mobile application is implemented to generate OTP for ATMs which increases the overall system defense. A chaotic encryption algorithm based on a key sequence, generated by a sequence of logistic maps and sequences of states of (LFSR) is also used to increase the security of iris image transmission over the internet. three publicly available datasets, namely Phoenix, UBIRIS. V1 and CASIA V4 Interval, captured using different modes of illumination and cameras, are used in experiments. The proposed model shows high and competitive results concerning the accuracy of recognition rate and training time. The average accuracy of the recognition rate obtained by the proposed model is 99.33% for overall datasets. The training time of the proposed model was 17, 22, and 25 min with Phoenix, UBIRIS. V1 and CASIA V4 datasets, respectively. Also, it shows a weak drop in recognition accuracy rates in case of noised iris images, therefore it is robust against noise interference due to sensing devices, bad iris acquisition due to system user interactions, or other client-end system attacks.

## Supplemental Information

10.7717/peerj-cs.381/supp-1 Supplemental Information 1 Code. NetBeans, Pycharm and Matlab software was used for Java, Python and Matlab codes respectively.

10.7717/peerj-cs.381/supp-2 Supplemental Information 2 Codes (TXT format). NetBeans, Pycharm and Matlab software was used for Java, Python and Matlab codes respectively.

## Additional Information and Declarations

### Data Availability

The following information was supplied regarding data availability: Data is available at the UBIRIS.v1 database (http://iris.di.ubi.pt/index_arquivos/Page374.html) and the Phoenix database (http://phoenix.inf.upol.cz/iris/). Code is available in the Supplemental Files.
